# Cbl-c Ubiquitin Ligase Activity Is Increased via the Interaction of Its RING Finger Domain with a LIM Domain of the Paxillin Homolog, Hic 5

**DOI:** 10.1371/journal.pone.0049428

**Published:** 2012-11-07

**Authors:** Philip E. Ryan, Stephen C. Kales, Rajgopal Yadavalli, Marion M. Nau, Han Zhang, Stanley Lipkowitz

**Affiliations:** 1 Laboratory of Cellular and Molecular Biology, Center for Cancer Research, National Cancer Institute, National Institutes of Health, Bethesda, Maryland, United States of America; 2 The George Washington University, Institute for Biomedical Sciences, Washington, District of Columbia, United States of America; George Washington University, United States of America

## Abstract

Cbl proteins (Cbl, Cbl-b and Cbl-c) are ubiquitin ligases that are critical regulators of tyrosine kinase signaling. In this study we identify a new Cbl-c interacting protein, Hydrogen peroxide Induced Construct 5 (Hic-5). The two proteins interact through a novel interaction mediated by the RING finger of Cbl-c and the LIM2 domain of Hic-5. Further, this interaction is mediated and dependent on specific zinc coordinating complexes within the RING finger and LIM domain. Binding of Hic-5 to Cbl-c leads to an increase in the ubiquitin ligase activity of Cbl-c once Cbl-c has been activated by Src phosphorylation or through an activating phosphomimetic mutation. In addition, co-transfection of Hic-5 with Cbl-c leads to an increase in Cbl-c mediated ubiquitination of the EGFR. These data suggest that Hic-5 enhances Cbl-c ubiquitin ligase activity once Cbl-c has been phosphorylated and activated. Interactions between heterologous RING fingers have been shown to activate E3s. This is the first demonstration of enhancement of ubiquitin ligase activity of a RING finger ubiquitin ligase by the direct interaction of a LIM zinc coordinating domain.

## Introduction

The covalent attachment of ubiquitin to proteins (known as ubiquitination or ubiquitylation) plays a fundamental role in regulating diverse cell processes including protein degradation, membrane protein trafficking, protein localization, enzyme activation, and DNA repair (reviewed in [1], [2]). Covalent attachment of ubiquitin to a substrate occurs through a sequential activation and conjugation of ubiquitin to a target protein by a series of three reactions [1], [2]. This is initiated by the ATP-dependent covalent attachment of the ubiquitin molecule to the active site cysteine of the ubiquitin activating enzyme (E1). The ubiquitin molecule is then transferred to via a transesterificaiton reaction to the active site cysteine on an ubiquitin-conjugating enzyme (E2). Subsequently, the E2 interacts directly with an ubiquitin ligase (E3) which facilitates the transfer of the ubiquitin molecule to the substrate. RING finger (RF) proteins constitute the majority of E3s, and accordingly they are fundamental regulators of many key cellular processes [3], [4]. The RF is comprised of ∼40–60 amino acids that form a coordination complex with two zinc ions [3], [4], [5]. The RF interacts with the E2 to mediate transfer of ubiquitin from the active site of the E2 in most cases to an acceptor lysine on target proteins or the growing end of ubiquitin chains. The E3 activity of RF proteins is regulated by covalent modifications of the substrate (*e.g.*, phosphorylation) [6], [7], homo- and heterodimerization mediated by the RF domains (*e.g.,* Mdm2 homodimers, Mdm2/MdmX heterodimers, and BARD1/BRCA1 heterodimers) [8], [9], [10], covalent modification of the RF protein (*e.g.,* phosphorylation and sumoylation) [7], [11], [12], [13], [14], and interaction with non-RF proteins (*e.g.,* MAGE proteins) [15].

Cbl proteins are RF E3s that negatively regulate signaling by many tyrosine kinases (*e.g.,* EGFR, Met, and Src) and tyrosine kinase-dependent pathways (*e.g.*, T-cell receptor). There are three mammalian Cbl proteins: Cbl (a.k.a., c-Cbl, Cbl2, and RNF55), Cbl-b (a.k.a., RNF56), and Cbl-c (a.k.a., Cbl-3, Cbl-SL, and RNF57) [16], [17] (We have used the HUGO nomenclature for the Cbl proteins. The nomenclature is as follows: Cbl refers to the first mammalian family member identified (a.k.a. c-Cbl, Cbl2, and RNF55); Cbl-b refers to the second mammalian Cbl protein identified (a.k.a. RNF56); Cbl-c refers to the third Cbl protein identified (a.k.a. Cbl-3, Cbl-SL, RNF57)). Cbl proteins have a highly conserved N-terminus consisting of a tyrosine kinase binding (TKB) domain that binds to specific phosphorylated tyrosines on substrates, a catalytic RF domain, and an alpha helical linker region separating the TKB and RF domains [16], [17]. The C-termini of the proteins are less highly conserved, although all three mammalian Cbl proteins have a proline rich (PR) region that mediates interaction with SH3-domain containing proteins. Cbl and Cbl-b have ubiquitin associated (UBA) domains at their C-termini that mediate homodimerization or ubiquitin binding, respectively. The E3 activity of the Cbl proteins is negatively regulated by the N-terminus of the proteins and activity is increased upon phosphorylation of a tyrosine in the linker region [11], [14].

While Cbl and Cbl-b have been well-studied and characterized, less is known about Cbl-c. Cbl-c is expressed exclusively in epithelial cells while Cbl and Cbl-b are widely expressed in mammalian tissues [18], [19], [20], [21]. Like Cbl and Cbl-b, the N-terminus of Cbl-c is composed of the highly conserved TKB, RF, and linker region [17], [19]. The C-terminus of the Cbl-c protein diverges from the other two Cbl proteins by having a shorter PR domain and lacking a UBA domain. Cbl-c, like Cbl and Cbl-b, is a functional E3 that can ubiquitinate and downregulate the EGFR, v-Src, and RET in cells [7], [19], [22], [23]. Mice null for Cbl and Cbl-b have clear immunological and hematological defects that help to define their physiological roles, but mice null for Cbl-c are viable and fertile with no clear abnormality [18], [24], [25], [26], [27]. Thus the physiological role of Cbl-c is not clear.

To elucidate the function of Cbl-c, we sought to identify Cbl-c interacting proteins utilizing a yeast two-hybrid screen and identified Hic-5 (a.k.a., TGFb1I1, ARA55) [28], [29] as a binding partner. Hic-5 is a member of the LIM domain containing paxillin family and the mRNA is widely expressed in normal tissues including epithelial tissues such as the lung, liver, and kidney and the mRNA is expressed in normal epithelial cells in culture [29], [30]. Interestingly, the mRNA is low or absent in transformed cells from hematopoietic and epithelial malignancies [29]. Hic-5 has been shown to be involved in many events involving cytoskeletal rearrangements, including focal adhesion formation, nuclear/cytoplasmic transport, cell migration, myotube formation, and epithelial to mesenchymal transition [29], [31], [32], [33], [34], [35], [36], [37], [38]. The Cbl-c/Hic-5 interaction is mediated by the RF of Cbl-c and the second LIM domain of Hic-5. Importantly, we established that Hic-5 enhances the E3 activity of Cbl-c both *in vitro* and in cells. Thus we have identified a novel interaction between two distinct zinc coordinating structures leading to enhancement of the Cbl-c RF E3 activity.

## Materials and Methods

### Materials

Dulbecco’s modified Eagle’s medium (DMEM), fetal bovine serum (FBS), penicillin, and streptomycin sulfate were obtained from Invitrogen (Carlsbad, CA). Dulbecco’s phosphate buffered saline (DPBS) was purchased from Mediatech Inc. (Herndon, VA). Recombinant human EGF was purchased from BD Biosciences, Inc. (San Jose, CA). Tissue culture plasticware and other laboratory consumables were purchased from commercial sources.

### Yeast Two-Hybrid Screening

Yeast two-hybrid screening was carried out at Myriad Genetics (Salt Lake City, UT) using a partial (amino acids 360–474) or a full length Cbl-c as the bait with a mating-based method. The Cbl-c cDNAs were cloned into pGBT.superB creating an open reading frame for Cbl-c fused to the GAL4 DNA-binding domain. The bait plasmid was introduced into Myriad’s ProNet yeast strain PNY200 (MATα *ura*3-52 *ade*2-101 *trp*1-901 *his*3-Δ200 *leu*2-3112 *gal*4Δ *gal*80Δ). The bait yeast cells were allowed to mate with Myriad’s ProNet MATa yeast cells, BK100 (MATα *ura*3-52 *trp*1-901 *his*3-Δ200 *leu*2-3112 *gal*4Δ *gal*80Δ GAL2-ADE2 LYS2::GAL1-HIS3 *met*2::GAL7-*lac*Z) containing three independent cDNA libraries from human breast/prostate cancer, spleen and brain. After mating, at least 5 million diploid yeast cells were obtained from each library and selected on His- and Ade-lacking medium. The auxotrophy is suppressed if the bait and prey proteins interact. The prey plasmids were isolated from the positive colonies, and the interaction was confirmed by expression of third reporter gene (lacZ). cDNAs in the positive prey plasmids were sequenced.

### Expression Constructs

The expression plasmids for full length HA-epitope tagged Cbl, Cbl-b, and Cbl-c, as well as Cbl-c Short and the control vector (pCEFL) have been described previously [19], [39]. HA-epitope deletion constructs for Cbl-c were created using PCR (ΔPro, ΔCT and ΔRF) or site-directed mutatgenesis (ΔCT2) to introduce a stop codon using Quick Change Kit (Stratagene, La Jolla, CA). HA-epitope Cbl-c mutants (C351A and C366A) were generated by site-directed mutagenesis using Quick Change Kit (Stratagene, La Jolla, CA). N-terminus tagged YFP-Cbl-c was constructed by first cloning Cbl-c ORF into the Hind III and Kpn I sites of pEGFP C3 (Clonetech, Mountain View, CA). The GFP protein was replaced with the YFP protein by cloning the YFP protein into the Age I and BsrG I sites of GFP. The pCMV-Hic-5 expression vector was obtained from Thermo Fisher Scientific/Open Biosystems (Huntsville, AL). Site directed mutagenesis of pCMV-Hic-5 to generate the C287A and C313A mutants was performed using the Quick Change Kit (Stratagene, La Jolla, CA). N-terminus tagged Hic5-eCFP in pReceiver-M3 was purchased from GeneCopoeia (EX-U1273-M32, Rockville, MD). The GFP expression plasmid used as a loading control for transfections (pCDNA3.1/zeo) was obtained from Invitrogen (Carlsbad, CA). The GST-tagged Cbl-c RF was created by PCR amplification of the Cbl-c RF and subsequent cloning of the PCR product into the 5′ BamHI and 3′ EcoRI restriction sites of pGEX-5X-1. His-tagged constructs for Cbl-c, Cbl-c Y341E, Hic-5, Hic-5 C287A, Hic-5 LIM2, and Hic-5 LIM2 C287A were created by amplifying the appropriate sequences using PCR and cloning the PCR products into the pTrc-HisA vector at the XhoI and HindIII restriction sites. pTrc-HisA was purchased from Invitrogen (Carlsbad, CA). pET15b-UbcH5b was a provided by Dr. Allan Weissman. All of the constructs were confirmed by DNA sequencing.

### Cell Culture and Transfections

Human embryonic kidney HEK293T, CFPAC-1, and HeLa cells were obtained from ATCC and maintained in culture in DMEM supplemented with 10% FBS, 100 U/ml penicillin, and 100 µg/ml streptomycin sulfate. HEK293T cells were transfected using calcium phosphate (Profection; Promega Corp., Madison, WI). Following transfection cells were grown 48 h prior to the preparation of cell lysates. CFPAC-1 and HeLa cells were transfected with Lipofectamine 2000 (Invitrogen, Carlsbad, CA). Transfections were allowed to incubate 18 h prior to rescue, and cells were grown an additional 48 h before being harvested.

### Immunoblotting and Immunoprecipitation

To harvest proteins, cells were washed twice in ice-cold DPBS containing 200 µM sodium orthovanadate (Fisher Chemicals, Fairlawn, NJ) and then lysed in ice-cold lysis buffer (10 mM Tris-HCl pH 7.5, 150 mM NaCl, 5 mM EDTA, 1% Triton X-100, 10% glycerol, 100 mM iodoacetamide (Sigma-Aldrich Corp., St. Louis, MO), 2 mM sodium orthovanadate, and protease inhibitors (Complete tabs®, Roche Diagnostics Corp., Indianapolis, IN). The lysates were cleared of debris by centrifugation at 16,000 ×*g* for 15 min at 4°C. Supernatant protein concentrations were determined using a BioRad protein assay (BioRad, Hercules, CA). For immunoblotting, lysates (2 µg protein/µl) were boiled in loading buffer (62.5 mM Tris-HCl pH 6.8, 10% glycerol, 2% SDS, 1 mg/ml bromphenol blue, 0.3573 M β-mercaptoethanol) for 5 min. For immunoprecipitation, transfected HEK293T lysates containing 200 µg protein were incubated with either a rabbit polyclonal anti-Hic-5 antibody (4914; Cell Signaling Technology Inc., Beverly, MA), mouse monoclonal anti-EGFR antibody (GR13; EMD Biosciences, Philadelphia, PA) and Protein A/G+ agarose beads (2003; Santa Cruz Biotechnology, Santa Cruz, CA) or HA-affinity matrix (11815016001; Roche Diagnostics Corp., Indianapolis, IN) overnight at 4°C with tumbling. Immune complexes were washed five times in cold lysis buffer, resuspended in 2 × loading buffer and boiled for 5 min. The proteins were resolved by SDS-PAGE and transferred to nitrocellulose membranes (Protran BA85; Whatman, Sanford, MA). Immunoprecipitation from HeLa and CFPAC-1 lysates containing 2000 µg protein were incubated with rabbit polycolonal anti-Cbl-c antibody (Rockland, Info) and Protein A/G + agarose beads overnight at 4°C with tumbling. Whole IPs were run over spin columns (Thermo Scientific, Rockford, IL) and washed with 500 µls of lysis buffer a total of three times. Proteins were eluted with 30 ml Non-Reducing Lane Marker Sample Buffer (Thermo Scientific, Rockford, IL). Pull downs of GST tagged proteins were performed by incubating 300 µg of cell lysate with 35 µl of glutathione sepharose beads (GE Healthcare, Pascataway, NJ) overnight at 4°C with tumbling. Pull downs were washed five times with 750 µl of lysis buffer and the precipitated proteins were resuspended in loading buffer and boiled for 5 min. Membranes were probed with either mouse monoclonal anti-Hic-5 antibody (611164; BD Transduction Laboratories, Franklin Lakes, NJ), Horseradish peroxidase linked mouse monoclonal anti-HA (3F10; Roche Diagnostics Corp., Indianapolis, IN), rabbit monoclonal anti-GFP (SC8334; Santa Cruz Biotechnology, Santa Cruz, CA), rabbit polyclonal anti-phospho-Src (2105; Cell Signaling, Danvers, MA), rabbit polyclonal anti-EGFR (2232; Cell Signaling, Danvers, MA), or anti-phosphotyrosine 4G10 Platinum (0501050; EMD Millipore Corp, Billerica, MA). Horseradish peroxidase linked donkey anti-rabbit IgG (NA934V; GE Healthcare, Piscataway, NJ), Horseradish peroxidase linked donkey anti-mouse IgG (NA931: GE Healthcare, Piscataway, NJ), or rabbit anti-goat (SC2768; Santa Cruz Biotechnology, Santa Cruz, CA) immunoglobulin was used with SuperSignal (Pierce Biotechnology Inc., Rockford, IL) to visualize the blots.

### Confocal Microscopy

HeLa cells (2×10^5^/well) were grown in a 6-well tissue culture dish in DMEM supplemented with 10% FBS, 100 U/ml penicillin, and 100 µg/ml streptomycin sulfate. 24 h after plating, cells were transiently transfected with YFP-Cbl-c and CFP-Hic-5 plasmids individually or together (2.0 ug DNA per well) using Lipofectamine-2000 reagent (Invitrogen, Carlsbad, CA) according to the manufacturer recommendations. 48 h post-transfection, cells were trypsinized and 2×10^4^cells were seeded on each well of 4-chambered coverglass slides (Lab-Tek, Scotts Valley, CA). Cells were rinsed twice with PBS and fixed in 4% paraformaldehyde for 10 min at room temperature. HeLa cells expressing YFP-Cbl-c and CFP-Hic-5 were imaged directly after fixation. For immunostaining focal adhesions, cells were permeabilized with PBS containing 0.1% saponin and 10% FBS for 5 min and incubated for 1 at room temperature or overnight at 4°C with the monoclonal anti-vinculin antibody (V9131; Sigma-Aldrich Corp., St. Louis, MO**)** at a 1∶400 dilution. The antibody was diluted in PBS containing 0.1% saponin and 10% FBS and cells were washed using the same buffer twice for 10 min. The cells were then incubated with Donkey anti-mouse Cy3-labeled secondary antibody (165–150; Jackson ImmunoResearch Laboratories, Inc., West Grove, PA) for 1 h at room temperature. The slides were washed twice in PBS–0.05% saponin with 10% FBS and once in PBS. The cells were examined using a Zeiss LSM 510 META confocal microscope with a 63× /1.3 NA immersion-oil Plan-Neofluar objective (Carl Zeiss, Inc., Thornwood, NY). All the images were collected using the same pinhole, offset gain and amplifier values below saturation. The imaris software (version 6.1.5; Bitplane Scientific Software, South Windsor, CT) was used for colocalization analysis, and Pearson’s correlation coefficient (PCC) was calculated for each image analysis on 5–10 cells for each combination.

### *In vitro* E3 Assays

*In vitro* E3 assays were performed in 30 µl reactions containing 50mM Tris-HCl pH7.5, 0.2 M ATP, 0.5 mM MgCl_2_, 0.1 mM DTT, and 1 mM phosphocreatine di(tris)salt (Sigma, St. Louis, MO), 15U creatine phophoskinase (EMD Biosciences, San Diego, CA), 50 ng purified recombinant rabbit ubiquitin-activating enzyme (662072; E1, EMD Biosciences, San Diego, CA), 0.5 µg ubiquitin (U100H; Boston Biochem, Cambridge, MA) and 1 µl of a crude bacterial lysate containing recombinant human UbcH5b (E2) [5], [14]. GST-tagged Cbl proteins coupled to GSH-Sepharose beads or purified soluble Cbl-c were added to the reaction and assays were performed at 30°C, shaking at 1000 RPM. General incubation time was 1 h unless stated otherwise. Reactions were stopped by addition of SDS-PAGE protein loading buffer and boiling for 5 min. Where stated, 100 ng active Src (14–236; Millipore, Lake Placid, NY) was added to the reaction. Where stated, approximately 200nM Hic-5, Hic-5 C287A, or Hic-5 LIM2 was added to the reaction. Samples were resolved using SDS/PAGE and analyzed by immunoblots using anti-ubiquitin (Z0458, DAKO North America, Inc. Carpinteria, CA).

### Production and Purification of His Proteins

Bacterial expression vectors were transformed in Rosetta bacteria (Invitrogen, Carlsbad, CA). Selected colonies were grown in LB broth with 100 µg/ml ampicillin selection. All His constructs were grown in 100 ml overnight cultures, diluted 1/10 in 900 ml of LB with 100 µg/ml ampicillin and grown at 37°C for 1 h. Protein production was induced with 2 mM isopropyl β-d-thiogalactoside at 37°C for 4 h. The culture was separated into 100 ml aliquots and pelleted by centrifugation. Extraction of His-tagged proteins from insoluble pellets was modified from a method previously described [40], [41]. Cell pellets were resuspended in 10 ml of sonication buffer (20 mM Tris pH 8.0, 0.3 M NaCl, 1 mM EDTA, 1 mg/ml lysozyme) and incubated for 30 min on ice. Resuspended pellets were sonicated for 12 sec a total of 12 times and transferred to 1.5 ml microcentrifuge tubes and centrifuged at 10,000×g at 4°C for 10 min. Supernantants were pooled and saved. The His-tagged Cbl-c proteins were contained in the insoluble pellet. The individual pellets were washed three times in wash buffer (20 mM Tris pH 8.0, 1% Triton X-100, 5 mM EDTA) with centrifugation at 10,000×g at 4°C for 15 m between washes. Pellets were combined and solubilized in 1 ml lysis buffer (0.3% N-lauroyl sarcosine, 50 mM CAPS buffer pH 11.0, 0.3 M NaCl) at room temperature, rotated for 20 min and centrifuged 15 min at 10,000 g. The supernatant was removed and any remaining pellet was resuspended and treated as described above until no pellet remained after centrifugation. Combined supernatants were incubated with 1-ml slurry of Ni-NTA resin previously washed with lysis buffer (Qiagen, Valencia, CA) and rocked overnight at 4°C. The combined resin and sample were applied to a column, washed with lysis buffer plus 5 mM imidazole two times, 20 mM imidazole once and eluted with 200 mM imidazole. Samples were evaluated by SDS-PAGE and staining with GelCode Blue Stain Reagent stained (Thermo Fischer Scientific, Rockford, IL). Proteins were dialyzed into 50 mM Bis-Tris pH 6.0, 20 mM NaCl, 10 uM ZnCl) using Fast Dialyzer chambers and RCIK membranes from Harvard Aparatus (Holliston, MA).

### Production and Purification of GST Proteins

Bacterial expression vectors were transformed in Rosetta bacteria. Selected colonies were grown in LB broth containing 100 µg/ml ampicillin. GST-Cbl-c RF constructs were grown in 10 ml overnight cultures and then diluted 1/10 in 90 ml of LB and grown at 37°C for 1 h. Protein production was induced with 2 mM isopropyl β-d-thiogalactoside at 37°C for 4 h. Cell pellets were resuspended in 5-ml of lysis buffer (50 mM Tris pH 8, 1 mM EDTA, 1% Triton-X 100, 5 mM DTT), sonicated and clarified by centrifugation. A 50% slurry of 300 µl of Glutathione Sepharose 4B (Amersham, Piscataway, NJ) was added to clarified lysate and incubated with rocking overnight at 4°C. Beads were pelleted by centrifugation and washed 5 times in lysis buffer and then 5 times in cold PBS. Proteins bound to GSH-beads were resuspended to a 50% slurry in PBS for storage.

## Results

### Yeast Two-hybrid Identifies Hic-5 as a Cbl-c Interacting Protein

**Figure 1:**
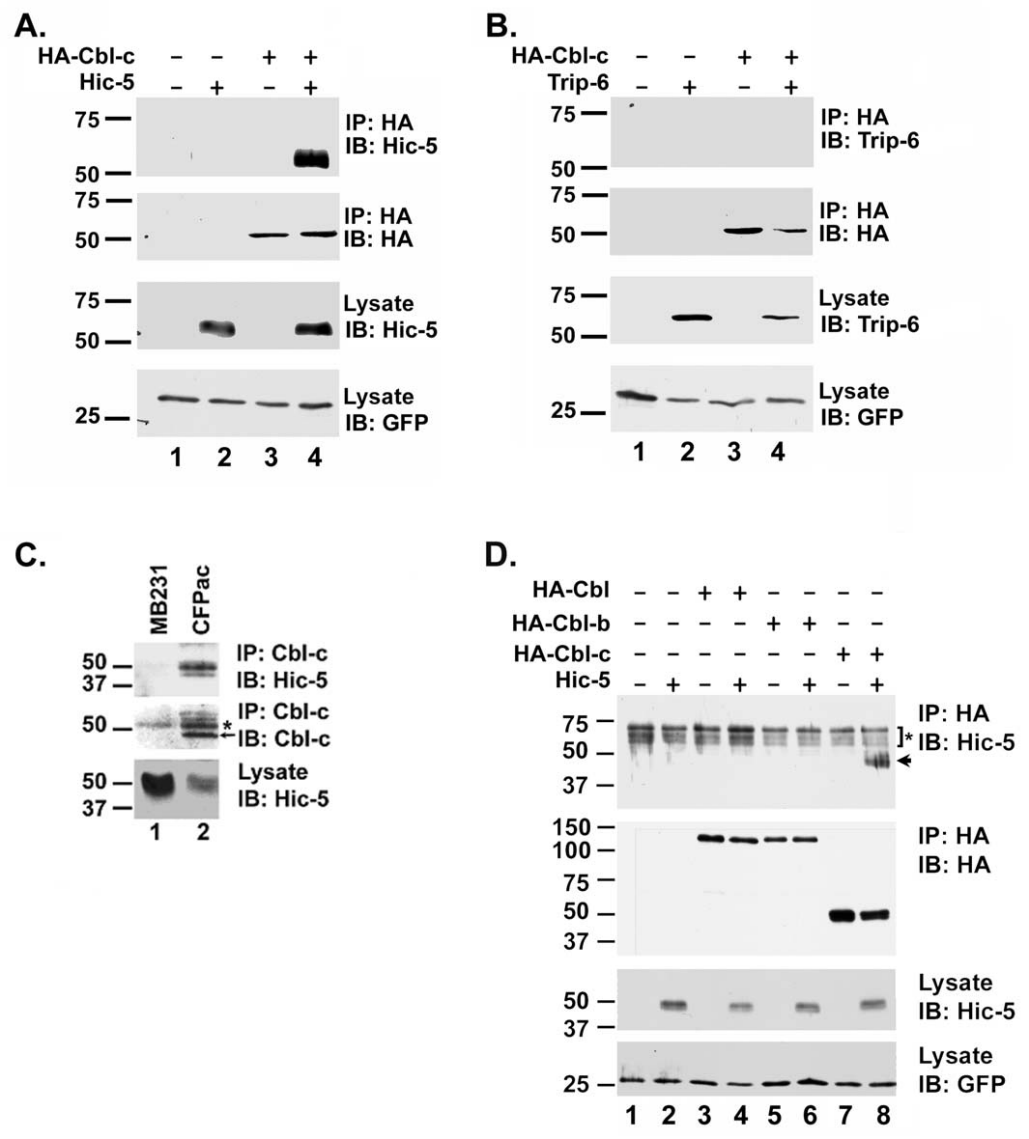
Hic-5 interacts with Cbl-c. **A.** HEK293T cells were transfected with HA-Cbl-c alone, Hic-5, or the combination of HA-Cbl-c and Hic-5 as indicated above panels. **B.** HEK293T cells were transfected with HA-Cbl-c alone, Trip-6, or the combination of HA-Cbl-c and Trip-6 as indicated above panels. In A and B, HA-Cbl-c was immunoprecipitated from the cell lysates. Immunoprecipitates (IP) or cell lysates (lysate) were immunoblotted (IB) as indicated to the right of the panels. All transfections were balanced with empty vector controls. Green fluorescent protein (GFP) was transfected as a control for transfection efficiency and is shown as a loading control. **C.** Endogenous Cbl-c was immunoprecipitated from whole cell lysates from MB231 (which expresses Hic-5 but no Cbl-c) and CFPAC-1 (which expresses both Hic-5 and Cbl-c) cell lines and immunoblotted for Cbl-c and Hic-5. Immunoprecipitates (IP) or cell lysates (lysate) were immunoblotted (IB) as indicated to the right of the panels. The arrow indicates immunoprecipitated Cbl-c and the asterisk indicates the immunoglobulin heavy chain. **D.** HEK293T cells were transfected with HA-Cbl, HA-Cbl-b, HA-Cbl-c alone, Hic-5 alone, or each HA epitope tagged Cbl protein with Hic-5 as indicated above the panels. Cbl proteins were immunoprecipitated and immunoprecipitates (IP) or cell lysates (lysate) were immunoblotted (IB) as indicated to the right of the panels. The arrow indicates immunoprecipitated Hic-5 and the asterisk indicates an artifact band that includes the immunoglobulin heavy chain. MWs in kDa are shown to the left of the panels.

Using partial and full length constructs of Cbl-c as bait in a yeast two-hybrid screen of breast cancer/prostate cancer, spleen, and brain cDNA libraries, the LIM domain containing proteins Hic-5 and Trip-6 were identified as potential Cbl-c interacting proteins. Both Hic-5 and Trip-6 were identified when full length Cbl-c (amino acids 1–474) and a construct containing the last 114 amino acids (360–474) were used as bait. To confirm the interactions of Cbl-c with Hic-5 and Trip-6, full-length cDNAs of Hic-5 and Trip-6 were co-expressed with HA-epitope tagged Cbl-c in HEK293T cells. Immunoprecipitation of HA-Cbl-c showed co-immunoprecipitation of Hic-5 but not of Trip-6 (Fig. 1A and B). These data confirmed the interaction of Cbl-c with Hic-5. The interaction between the endogenous proteins was established by the demonstration of specific co-immunoprecipitation of Hic-5 from lysates from cells that express both proteins but not from cells that express high levels of endogenous Hic-5 but lack detectable Cbl-c using an anti-Cbl-c antibody (Fig. 1C). When immunoprecipitating Cbl-c from the CFPAC-1 cell line, we sometimes see higher molecular weight bands (see above asterisk in Fig. 1C). The identity of these bands is not known but could represent post-translationally modified forms of Cbl-c.

Cbl-c is the most divergent of the three mammalian Cbl proteins [17], [19]. To test the interaction of Hic-5 with each of the Cbl proteins, Hic-5 was co-expressed with HA-epitope tagged Cbl, Cbl-b, and Cbl-c in HEK293T cells, followed by immunoprecipitation of Cbl proteins with an anti-HA affinity matrix. Hic-5 co-precipitated with Cbl-c, but not with Cbl or Cbl-b, suggesting a selective interaction between Cbl-c and Hic-5 (Fig. 1D).

**Figure 2:**
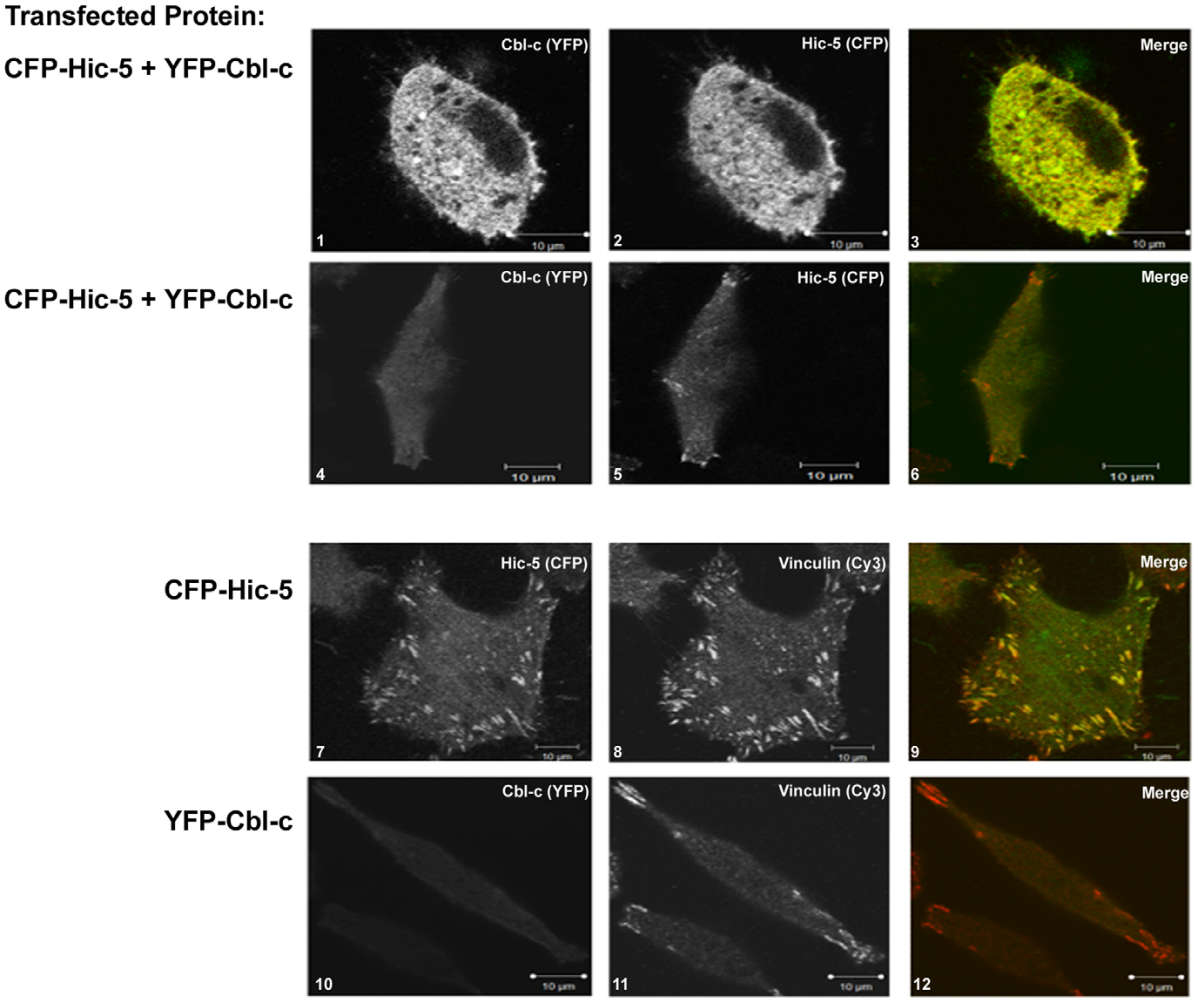
Hic-5 colocalizes with Cbl-c. HeLa cells were transfected with CFP-Hic-5 and/or YFP-Cbl-c, fixed, and imaged using a Zeiss LSM 510 META confocal microscope with a 63X /1.3 NA immersion-oil Plan-Neofluar objective as described in the methods section. Images for each protein are shown in grey scale and for merged images in color. Panels 1–3 demonstrate the diffuse localization of YFP-Cbl-c and CFP-Hic-5. Panels 4–6 demonstrate the punctate localization of CFP-Hic-5, but not YFP-Cbl-c. In the merged images (panels 3 and 6) YFP-Cbl-c is shown in green, CFP-Hic-5 is shown in red and the overlapping signal is shown in yellow. To demonstrate that the punctate distribution of CFP-Hic-5 represents localization to focal adhesions, cells were stained using an anti-vinculin antibody as a marker of focal adhesions. Panels 7–9 demonstrate the colocalization of CFP-Hic-5 and vinculin in the punctate structures, consistent with colocalization in focal adhesions. Panels 10–12 show no colocalization of YFP-Cbl-c with vinculin. In the merged images for the vinculin staining (panels 9 and 12) YFP-Cbl-c and CFP-Hic-5 are shown in green, vinculin is shown in red, and the overlapping signal is shown in yellow. The imaris software was used for colocalization analysis, and Pearson’s correlation coefficient (PCC) was calculated for each image analysis on 5–10 cells for each combination (see text for results).

Hic-5 has been described to localize to focal adhesions, the cytosol, and to the nucleus [42], [43]. To investigate the site of interaction between Hic-5 and Cbl-c, CFP-Hic-5 and YFP-Cbl-c were transfected into HeLa cells and visualized using confocal microscopy (Fig. 2). Cbl-c and Hic-5 overlapped in a diffuse pattern (Fig. 2, panels 1–3; Pearson’s Correlation Coefficient  =  0.70 +/–0.15). Hic-5 was also found in a punctate distribution on the basal surface of the cells consistent with its known localization to focal adhesions but Cbl-c was not localized to these structures (Fig. 2, panels 4–6; Pearson’s Correlation Coefficient  =  −0.06 +/−0.07). To confirm that the punctuate structures were focal adhesions, cells transfected with CFP-Hic-5 or YFP-Cbl-c were stained with an antibody for vinculin, a protein specific for focal adhesions. Hic-5 and vinculin colocalized to the punctuate structures confirming that these are focal adhesions (Fig. 2, panels 7–9; Pearson’s Correlation Coefficient  =  0.70 +/−0.23). No colocalization of Cbl-c and vinculin was found (Fig. 2, panels 10–12; Pearson’s Correlation Coefficient  =  −0.11 +/− 0.10).

The overlap of Cbl-c and Hic-5 in a diffuse compartment and the lack of colocalization of Cbl-c and Hic-5 in a clearly identifiable compartment (*e.g.,* focal adhesions) makes the determination of the site of their interaction inconclusive.

### Hic-5 Interacts with the RING Finger of Cbl-c

**Figure 3:**
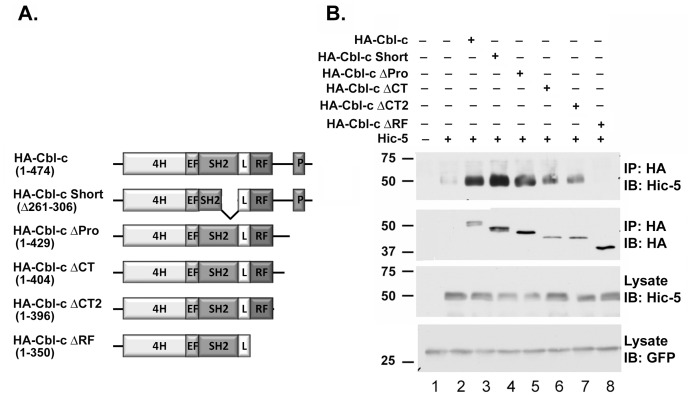
Hic-5 interacts with the RF of Cbl-c. **A.** Schematic diagram of Cbl-c constructs used to map the interaction with Hic-5. All constructs were HA-epitope tagged on the N-terminus. **B.** HEK293T cells were transfected with various HA- Cbl-c constructs and Hic-5 as indicated above panels. Immunoprecipitates (IP) or cell lysates (lysates) were immunoblotted (IB) as indicated to the right of the panels. All transfections were balanced with empty vector controls. GFP was transfected as a control for transfection efficiency and shown as a control for loading. MWs in kDa are shown to the left of the panels.

**Figure 4:**
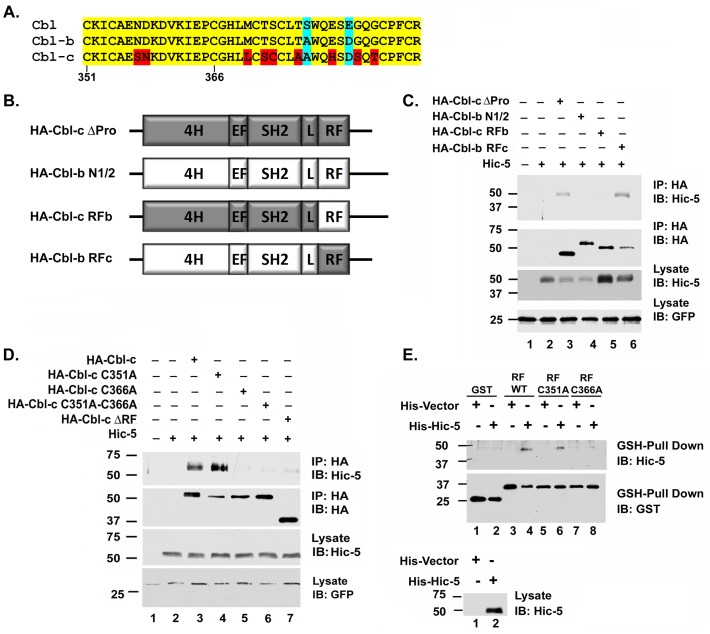
Mutation of the first zinc coordinating complex of the RF of Cbl-c disrupts Hic-5 binding. **A.** Sequence alignment of the RFs of Cbl, Cbl-b and Cbl-c. Amino acids in red show the nine amino acids that are divergent between the RF of Cbl-c and the RFs of Cbl and Cbl-b. The two positions highlighted in blue mark the amino acids shared between Cbl-c and Cbl-b that differ from Cbl. The two cysteines mutated to alanine in Figure 3D and E are indicated below the sequence. **B.** Schematic diagram of Cbl-c, Cbl-b and chimeric constructs with the swapped RF domains. Shaded boxes represent Cbl-c sequence. White boxes represent Cbl-b sequence. All constructs were HA-epitope tagged on the N-terminus. **C.** HEK293T cells were transfected with HA-Cbl-c, Cbl-b, or the chimeric proteins with swapped RF domains and Hic-5 as indicated above the panels. The Cbl proteins were immunoprecipiated with anti-HA beads and the immunoprecipitates (IP) or cell lysates (lysates) were immunoblotted (IB) as indicated to the right of the panels. All transfections were balanced with empty vector controls; GFP was transfected as a control for transfection efficiency and is shown as a control for loading. **D.** HEK293T cells were transfected with HA-Cbl-c, RF mutants of HA-Cbl-c, HA-Cbl-c ΔRF, and Hic-5 as indicated above the panels. The Cbl-c proteins were immunoprecipitated with anti-HA beads and the immunoprecipitates (IP) or cell lysates (lysates) were immunoblotted (IB) as indicated to the right of the panels. All transfections were balanced with empty vector controls; GFP was transfected as a control for transfection efficiency and is shown as a control for loading. **E.** Purified recombinant GST-tagged Cbl-c RF proteins (WT, C351A or C366A mutants) bound to GSH-sepharose beads were added to a bacterial lysate expressing recombinant His-tagged Hic-5 or empty His vector as indicated above the panel. Proteins were precipitated with GSH-sepharose beads and immunoblotted (IB) as indicated to the right of the panels. Expression of His-Hic-5 in the bacterial lysates is shown in the lower panel. MWs in kDa are shown to the left of the panels.

To map the site of the interaction of Cbl-c with Hic-5, a series of deletion mutations of Cbl-c were coexpressed with Hic-5 in HEK293T cells and assessed for co-immunoprecipitation with Hic-5 (Fig. 3). The deletion of the last 124 amino acids of Cbl-c including the RF abrogated binding to Hic-5 (Fig. 3B, lane 8). This is consistent with the identification of Hic-5 in the yeast two-hybrid screen by a Cbl-c bait containing the last 114 amino acids. In contrast, successive deletions of the C-terminus up to the RF did not affect binding of Cbl-c to Hic-5A (Fig. 3B, lanes 5–7). Also, a naturally occurring splice variant (Cbl-c Short) that deletes the second half of the SH2 domain of Cbl-c (and abrogates binding to tyrosine phosphorylated proteins) did not affect binding to Hic-5 (Fig. 3B, lane 4). These results identified the RF as a critical domain for the interaction between Cbl-c and Hic-5. The RF of Cbl-c differs from the RF of Cbl and Cbl-b by nine of 40 amino acids (Fig. 4A). To further test the specificity of the interaction between Hic-5 and the RF of Cbl-c, chimeric proteins of Cbl-b containing the RF of Cbl-c and Cbl-c containing the RF of Cbl-b were expressed along with Hic-5 in HEK293T cells and their interactions assayed by co-precipitation (Fig. 4B and C). As controls, the non-chimeric versions of these constructs for Cbl-b and Cbl-c were tested (Fig. 4B and C). Hic-5 co-precipitated with Cbl-c and with the chimeric Cbl-b containing the Cbl-c RF but not with either Cbl-b or the chimeric protein of Cbl-c containing the Cbl-b RF. Together, these data indicated that the RF of Cbl-c is critical for the interaction with Hic-5.

The Cbl RF is a cross braced C3HC4 zinc coordinating motif [4], [44]. Using site-directed mutagenesis, we tested the effect of mutating the first cysteine in the first zinc coordinating complex (C351A) of the RF of Cbl-c, the first cysteine in the second zinc coordinating complex (C366A) in the RF of Cbl-c, or both on the ability of Cbl-c to interact with Hic-5. The Cbl-c and Hic-5 interaction was abrogated in the C366A mutant and in the double mutant but not with the C351A mutant (Fig. 4D, compare lanes 5 and 6 to lanes 3 and 4). This suggests that the second zinc coordination complex of the Cbl-c RF is necessary for the interaction. To further test the specificity of the interaction between the RF of Cbl-c and Hic-5, we performed pull down assays using a recombinant purified GST-tagged protein containing the isolated RF of Cbl-c (WT, C351A and C366A) and a bacterial lysate expressing recombinant His-tagged Hic-5. Hic-5 precipitated with GST-RF WT and GST-RF C351A mutant (Fig. 4E, lanes 4 and 6) but not with GST or GST-RF 366A (Fig. 4E, Lanes 2 and 8).

### Cbl-c Interacts with the LIM2 of Hic-5

**Figure 5:**
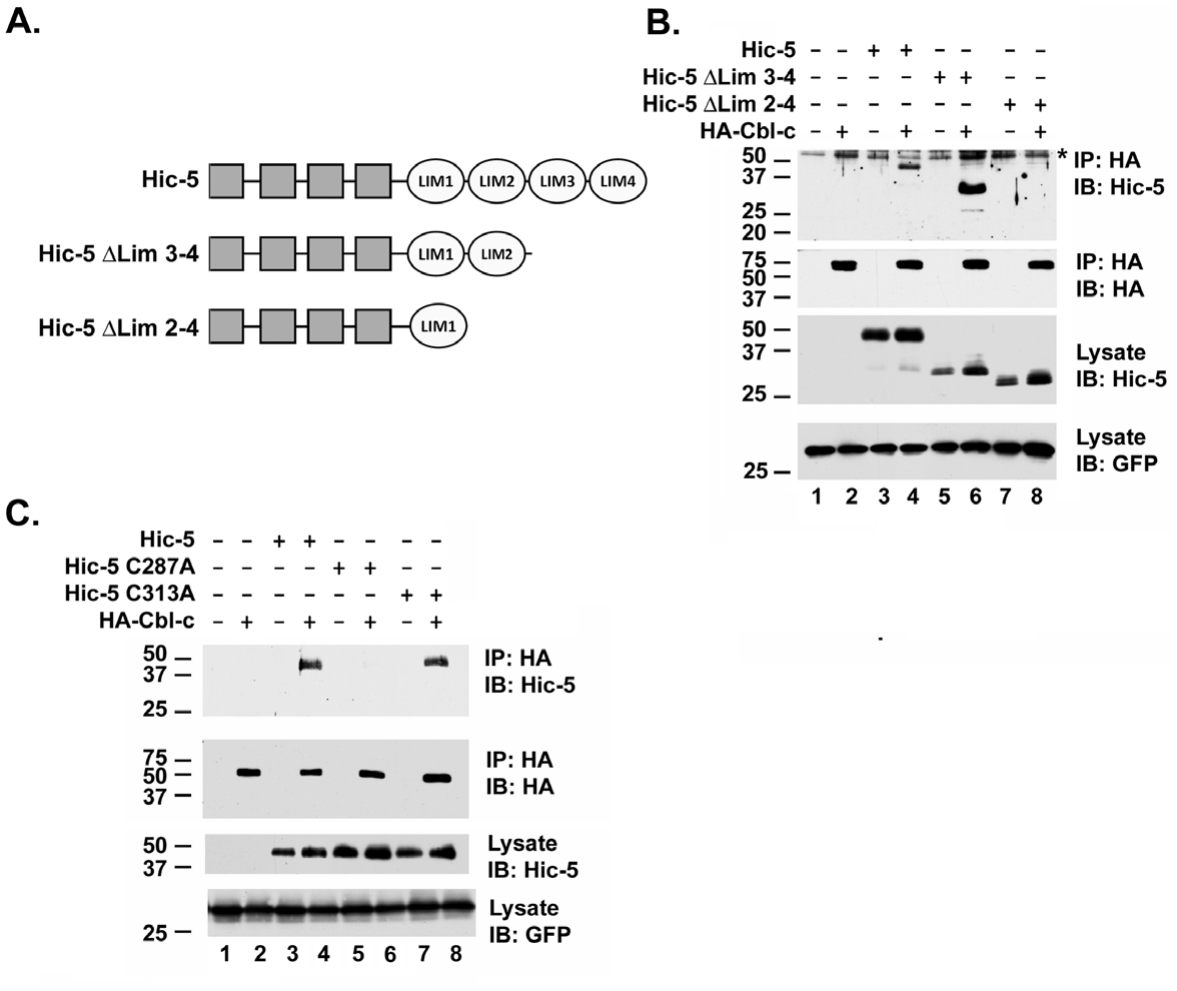
The second zinc coordinating complex of Hic-5 LIM2 is required for the interaction with Cbl-c. **A.** Schematic diagram of Hic-5 constructs used to map the interaction with Cbl-c. **B.** HEK293T cells were transfected with HA- Cbl-c and constructs for Hic-5 as indicated above the panel. HA-Cbl-c was immunoprecipitated with anti-HA beads and immunoprecipitates (IP) or cell lysates (lysate) were immunoblotted (IB) as indicated to the right of the panels. * indicates immunoglobulin. **C.** HEK293T cells were transfected with HA-Cbl-c along with WT or mutants of Hic-5 which disrupt either the first or second zinc coordinating complexes of LIM2 (C287A and C313A respectively) as indicated above the figure. HA-Cbl-c was immunoprecipitated with anti-HA beads and immunoprecipitates (IP) or cell lysates (lysate) were immunoblotted (IB) as indicated to the right of the panels. All transfections were balanced with empty vector controls; GFP was transfected as a control for transfection efficiency and is shown as a loading control. MWs in kDa are shown to the left of the panels.

Hic-5 is a member of the paxillin family of LIM proteins that are characterized by N-terminal LD protein-interacting domains and four C-terminal LIM domains [29], [30]. The minimal interacting region of Hic-5 identified by the Cbl-c baits (amino acids 125–316) included the second two LD domains and the first two LIM domains. Full length Hic-5 and a deletion mutant removing LIM domains 3 and 4 co-precipitated with Cbl-c (Fig. 5B, lanes 4 and 6) but deletion of LIM domains 2–4 abrogated the interaction with Cbl-c (Fig. 5B, lane 8). These data suggest that LIM domain 2 of Hic-5 is necessary for interaction with Cbl-c.

The Hic-5 LIM2 domain is a C2HC3D zinc finger [29]. While the RF and LIM domains are both zinc fingers that have two zinc coordinating complexes, they are structurally different and the LIM domains have no reported E3 activity [45]. For either structure, mutation of any of the zinc coordinating amino acids disrupts the coordination of the zinc atom [29], [44], [45]. Again, we used site-directed mutagenesis to mutate the first cysteine of the first zinc coordinating complex of LIM2 (C287A) or the first cysteine of the second zinc coordinating complex of LIM2 (C313A) in the full length Hic-5 protein. The interaction between Cbl-c and Hic-5 was disrupted by mutation of the first zinc coordinating complex of the Hic-5 LIM2 domain but not by mutation of the second zinc coordinating complex of the Hic-5 LIM2 domain (Fig. 5C, compare lane 6 to lane 4 and 8). These data coupled with those of the RF mutant data suggest a specific interaction between the region around the second zinc coordinating complex of the Cbl-c RF and the region around the first zinc coordinating complex of the second LIM domain of Hic-5.

### Hic-5 Enhances Cbl-c E3 Activity *in vitro*

**Figure 6:**
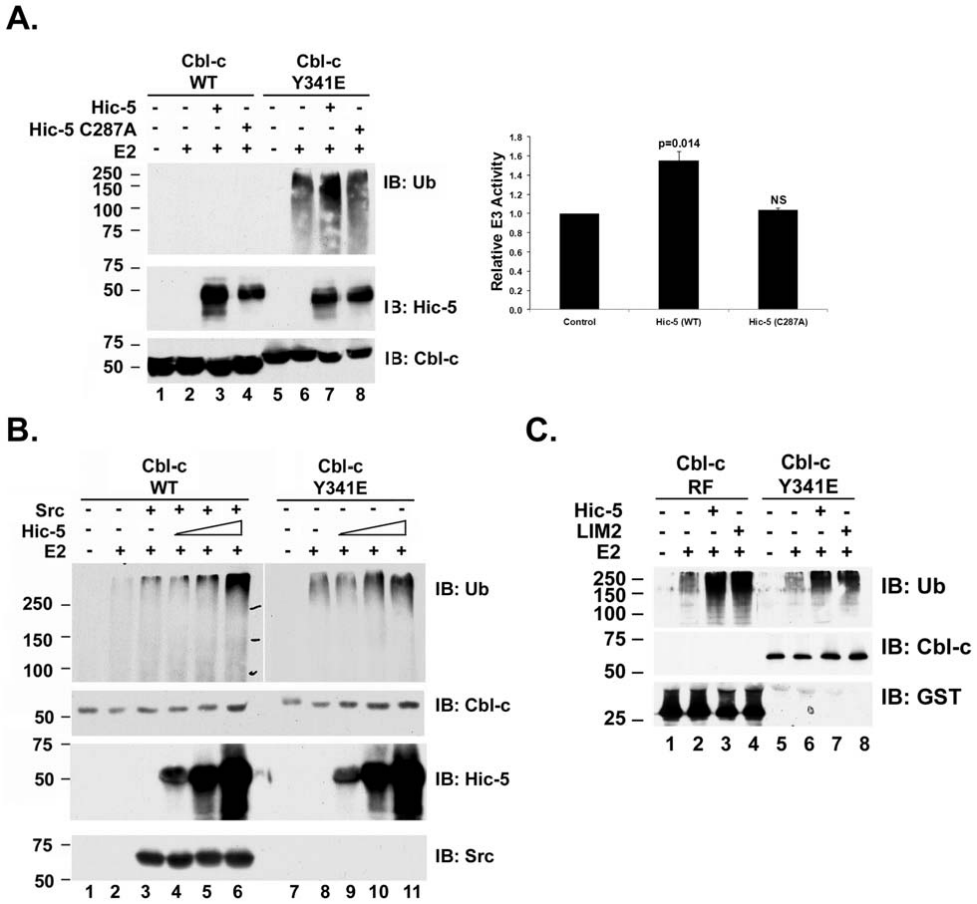
Hic-5 increases the E3 activity of Cbl-c *in vitro*. **A.** *In vitro* E3 assays were performed as described in the methods with the recombinant WT or activated (Y341E) His-Cbl-c constructs as labeled above panel. Recombinant purified His-Hic-5 WT, C287A were added where indicated. The data for the E3 activity of the Y341E Cbl-c mutant were quantified for three experiments and are displayed on the graph. Values represent the mean ubiquitination +/– SE relative to Y341 Cbl-c alone. The dotted line represents the level of ubiquitination by Y341E Cbl-c in the absence of Hic-5. Two tailed p-values using a paired T-test comparing the results for Y341 Cbl-c + Hic-5 (WT or C287A) to Cbl-c are shown on the graph (NS  =  not significant). **B.** E3 assays were performed with either WT or Y341E recombinant Cbl-c as described above. Increasing amounts of recombinant His-Hic-5 was added as indicated above the panel. Purified active Src was added where indicated. **C.** *In vitro* E3 assays were performed with recombinant GST-Cbl-c RF (GST-RF) or GST-Cbl-c Y341E as described above. Recombinant His-Hic-5 or the His-LIM2 domain of Hic-5(LIM2) were added as indicated above the panel. *In vitro* E3 assays were performed at 30°C for 40 m in the presence and absence of E2 and then immunoblotted (IB) as indicated. MW markers in kDa are shown to the left of the panels.

Cbl proteins are E3s whose activity depends on the RF [7]. Considering the unique nature of the interaction between Hic-5 and the RF domain of Cbl-c we tested whether this interaction would affect the E3 activity of Cbl-c. Using an *in vitro* E3 assay [14] His-tagged purified recombinant Cbl-c WT was incubated with recombinant E1, E2 and Ub in the presence or absence of purified Hic-5 or the non-binding Hic-5 C287A mutant. Under these conditions, there was no detectable E3 activity and Hic-5 did not lead to increased activity of WT Cbl-c (Fig. 6A, lanes 2–4). Previous work has demonstrated that phosphorylation or a phosphomimetic Y to E mutation of a tyrosine located on the alpha-helical linker that precedes the RF dramatically increases the activity of Cbl proteins when measured by *in vitro* E3 autoubiquitination assays [11], [14]. We next tested whether Hic-5 would affect the activity of a phosphomimetic mutant (Cbl-c Y341E). An E3 assay with Cbl-c Y341E either alone, with Hic-5 or with Hic-5 C287A showed that Hic-5 increased the autoubiquitination of Cbl-c Y341E while addition of Hic-5 containing the C287A mutation did not (Fig. 6A, compare lane 7 to lanes 6 and 8). Quantification of the data by densitometry for three experiments demonstrated that WT Hic-5 resulted in an approximately 1.5 fold increase in Cbl-c autoubiquitination while Hic-5 C287A did not significantly enhance Cbl-c autoubiquitination (Fig. 6A, graph). To further study the increase in Cbl-c E3 activity, we incubated Cbl-c Y341E with increasing concentrations of Hic-5 which resulted in a dose dependent increase in E3 activity (Fig. 6B, lanes 8–11). As previously shown [14], incubation of WT Cbl-c with Src results in a stimulation of E3 activity (Fig. 6B, compare lanes 2 and 3). The addition of Hic-5 resulted in a dose dependent stimulation of the E3 activity (Fig. 6B, compare lanes 4–6 to lane 3). These data suggest that Hic-5 can increase the activity of Cbl-c only after the E3 is activated by phosphorylation.

Previous work has demonstrated that deletion of the N-terminus of Cbl-c also enhances the E3 activity of Cbl-c and the isolated RF of Cbl-c has robust E3 activity [14]. Thus, we tested whether Hic-5 could increase the E3 activity of the isolated Cbl-c RF. Similar to the phosphomimetic Cbl-c Y341 E mutant, Hic-5 increased the autoubiquitination of the isolated RF of Cbl-c (Fig. 6C, compare lanes 3 and 7 to lanes 2 and 6, respectively). Also, we found that a purified recombinant protein containing only the LIM2 domain of Hic-5 enhanced the activity of either the Cbl-c RF or Cbl-c Y341E (Fig. 6C, lanes 4 and 8).

### Hic-5 Enhances Cbl-c Mediated Ubiquitination of EGFR

**Figure 7:**
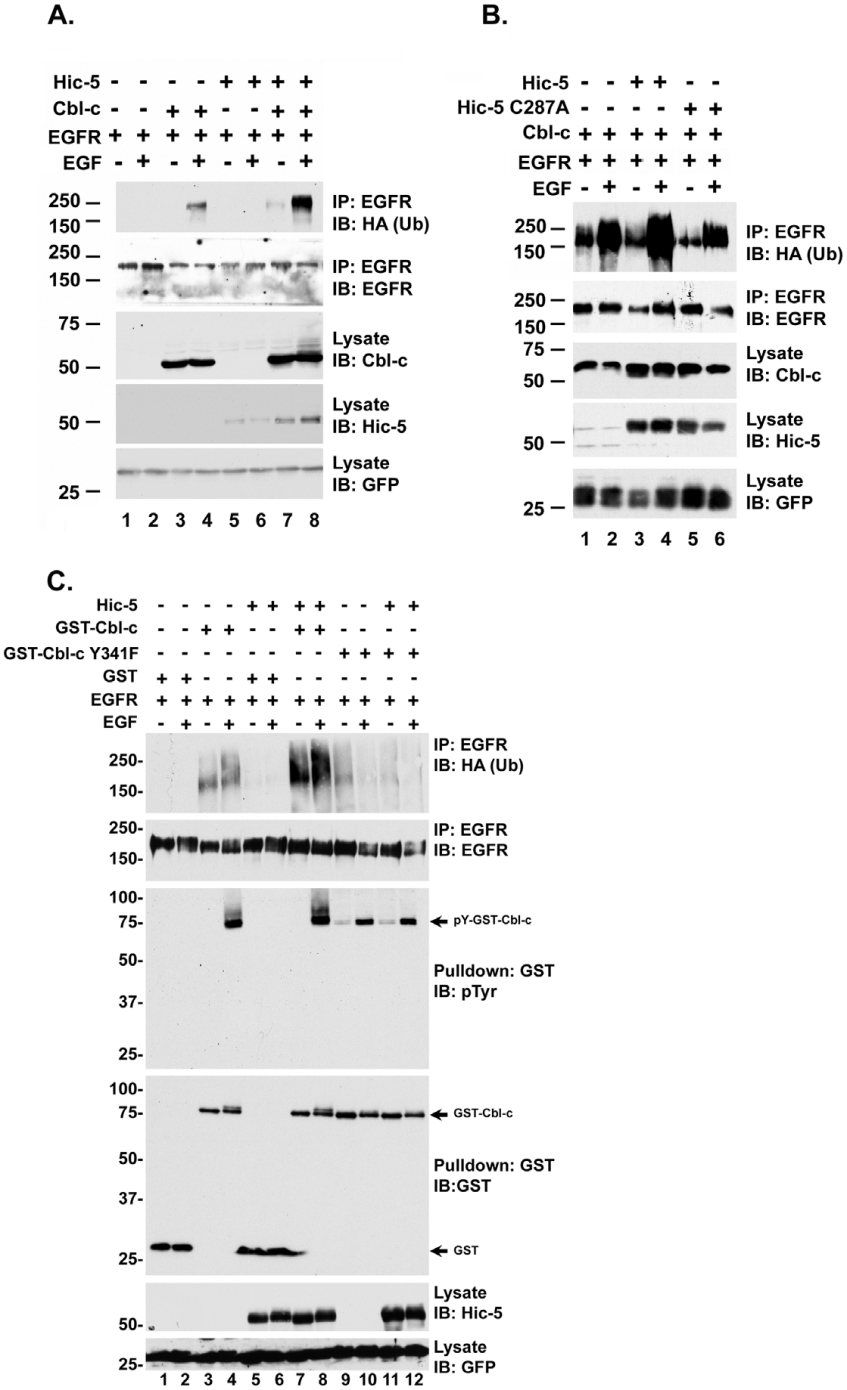
Hic-5 enhances Cbl-c mediated ubiquitination of EGFR in cells. **A.** HEK293T cells were transfected with EGFR, HA-epitope tagged ubiquitin, with and without Cbl-c in the presence and absence of Hic-5 as indicated above the panels. **B.** HEK293T cells were transfected with EGFR, HA-epitope tagged ubiquitin and Cbl-c alone and with either Hic-5 WT or Hic-5 C287A. **C.** HEK293T cells were transfected with EGFR, HA-epitope tagged ubiquitin, GST-Cbl-c, GST-Cbl-c Y341F, and Hic-5 WT as indicated. All transfections were performed in duplicate. Cells were serum starved for 18 h, and one plate of each pair was stimulated with 10 ng/ml EGF for 15 min. EGFR was immunoprecipitated and immunoprecipitates (IP) or cell lysates (lysates) were immunoblotted (IB) as indicated to the right of the panels. All transfections were balanced with empty vector controls; GFP was transfected as a control for transfection efficiency and shown as a loading control. MWs in kDa are shown to the left of the panels.

Ubiquitination of the activated EGFR is a well studied function of Cbl proteins [6], [7], [46], [47], [48]. To investigate whether the interaction between Cbl-c and Hic-5 results in increased E3 activity of Cbl-c in cells, we tested whether Hic-5 could affect the ability of Cbl-c to ubiquitinate the EGFR. HEK293T cells were transfected with the EGFR alone, with WT Hic-5 with and without WT Cbl-c. All transfections included an HA-epitope tagged ubiquitin. Ubiquitination of the EGFR was assessed by immunoprecipitation of the EGFR and immunoblotting with anti-HA. Cbl-c increased the ubiquitination of the EGFR upon EGF activation (Fig. 7A, compare lanes 2 and 4), and this was further increased by the co-expression of Hic-5 (Fig. 7A, lane 8). Subsequent co-expression studies using Hic-5 C287A in addition to WT Hic-5 showed that the non-binding Hic-5 C287A mutant did not increase the ubiquitination of EGFR by Cbl-c (Fig. 7B).

Hic-5 enhanced autoubiquitination of Cbl-c *in vitro* only when Cbl-c was activated by phosphorylation or by a phosphomimetic mutation of the linker tyrosine Y341 (Fig. 6). Previous work has shown that mutation of Y341 to phenylalanine (F) abrogates Cbl-c E3 activity [14]. To test whether activation of Cbl-c by phosphorylation on the linker tyrosine was required for Hic-5 to enhance the ubiquitination cells were transfected with EGFR +/– WT or Y341F GST-Cbl-c in the absence or presence of WT Hic-5 (Fig. 7C). Ubiquitination of EGFR by GST-Cbl-c was enhanced by Hic-5 (Fig. 7C, top panel, compare lanes 3 and 4 to lanes 7 and 8). EGF did not induce ubiquitination of the EGFR by GST-Cbl-c Y341F and Hic-5 did not enhance ubiquitination of EGFR by the mutant Cbl protein (Fig. 7C, top panel, lanes 9–12). GST pull down of the Cbl protein demonstrated that EGF induced significant tyrosine phosphorylaton of GST-Cbl-c, but this was decreased in GST-Cbl-c Y341F (Fig. 7C, third panel, compare lanes 4 and 8 to lanes 10 and 12). Interestingly, the phosphorylation of the WT Cbl-c protein was accompanied by a slower migrating band on the gel that was absent in the Y341F mutant Cbl-c (Fig. 7C, fourth panel, compare lanes 4 and 8 to lanes 10 and 12).

We often observe ubiquitination of unstimulated EGFR when the EGFR is overexpressed with a Cbl protein in 293T cells (*e.g.* see Fig. 7B, lane 1 or Fig. 7C, lane 3). Interestingly, the presence of Hic-5 increases the level of ubiquitination of the unstimulated EGFR in the presence of Cbl-c (Compare Fig. 7A or Fig 7C, lanes 3 and 7). We believe this is due to the presence of activated EGFR when the EGFR is overexpressed due either to endogenous ligand or forced proximity due to overexpression.

Overall, Hic-5 enhanced both autoubiquitination of activated Cbl-c *in vitro* and substrate ubiquitination by activated Cbl-c in a cellular model.

## Discussion

In this study we used a yeast two-hybrid screen to identify a new Cbl-c interacting protein, Hic-5 (Fig. 1). Hic-5 is a LIM containing protein in the paxillin family that consists of eight protein-protein interacting domains: four LD domains and four LIM domains [29], [30]. Hic-5 has been implicated in cytoskeletal function, as a mediator of epithelial to mesenchymal transition, cellular senescence, and differentiation [29], [31], [33], [34], [35], [36], [37], [38]. The nature of the interaction of Cbl-c and Hic-5 is unique. The interaction between the two proteins is mediated by the RF of Cbl-c and the LIM2 domain of Hic-5 (Figs. 2, 3, 4). Furthermore, we demonstrate that this interaction is mediated by specific zinc coordinating complexes within each of the specified domains (Figs. 3 and 4). The RF and LIM domains are each composed of two zinc coordinating complexes [30], [44], [45]. The disruption of the second zinc coordinating complex, but not the first, in the RF of Cbl-c resulted in the abrogation of the interaction with Hic-5. Disrupting the first zinc coordinating complex, but not the second, of the LIM2 domain of Hic-5 eliminates the interaction with Cbl-c. This suggests that the interaction between Hic-5 and Cbl-c is coordinated very specifically by the maintenance of these zinc finger structures. To our knowledge there have been no published reports of a RF-LIM domain interaction.

Hic-5 interacts with Cbl-c but not with the closely related proteins Cbl or Cbl-b (Fig. 1C). While the RF domains of the three mammalian Cbl proteins are highly conserved, the RF of Cbl-c is the most divergent of all of the Cbl proteins. Cbl and Cbl-b are identical at 38 of the 40 amino acids in the core RF domain and the two non-identical amino acids are conservative changes (Fig. 4A). By contrast, Cbl-c differs at nine amino acids from either Cbl or Cbl-b. Swapping the RF of Cbl-c with the RF of Cbl-b confirmed that the Hic-5 interaction is mediated only by the RF of Cbl-c (Fig. 4C). It is interesting that disruption of the second zinc coordinating complex of Cbl-c, but not the first, abrogates the interaction between Cbl-c and Hic-5. Six of the nine amino acid differences between the RF of Cbl-c and the RFs of Cbl and Cbl-b are in the loops between the first and second coordination complexes. Together these data suggest that the binding of Hic-5 to Cbl-c is mediated by the region surrounding the second zinc coordination complex.

Tyrosine phosphorylation of Cbl proteins by their substrates is necessary for E3 activity [7]. Previously we and others have shown that the N-terminus of Cbl-c inhibits E3 activity and that phosphorylation of tyrosine 341 in the linker region preceding the RF or deletion of the N-terminus leads to an increase in E3 activity [11], [14]. In this study we demonstrate that purified recombinant Hic-5, but not the non-binding C287A mutant, increases the autoubiquitination of Cbl-c which is activated by a phosphomimetic Y to E mutation of the linker tyrosine or the deletion of the N-terminus *in vitro* while having no measureable effect on an unactivated Cbl-c (Fig. 6).

For the most part, only E2 and RF proteins have been described to interact via the RF [3], [4]. Regulation of E3 activity by the interaction between two different RF domains has been described in a number of cases. For example, BRCA has weak E3 activity that is markedly stimulated by heterodimerization with BARD1 [8]. MDM2 heterodimerizes with the closely related RF protein, MDMX, resulting in increased ubiquitination of p53 and decreased autoubiquitination [9], [10]. In both cases, only one member of the dimer (BRCA1 for BRCA1/BARD and MDM2 for MDM2/MDMX) has demonstrated E3 activity while the second RF domain, without E3 activity of its own, serves to regulate the activity of the first RF domain [8], [9], [10]. RF mediated homodimerization of RF proteins has also been described [3]. For example, upon binding of SMAC or SMAC mimetics to cIAP1, the cIAP1 protein homodimerizes via the RF. This results in autoubiquitination and degradation of the cIAP1 protein [49]. The binding of Hic-5, a protein that is neither an E2 nor a RF protein, to the RF of an E3 appears to be a novel interaction. Interestingly, the interaction is mediated by the zinc coordinating LIM2 domain of Hic-5. Like the interactions described between the zinc coordinating RF of BARD1 and the RF of BRCA1, Hic-5 enhances E3 activity of the Cbl-c RF but Hic5 has no detectable E3 activity by itself.

The isolated LIM2 of Hic-5 is sufficient for this increased activity, and further, the isolated LIM2 can increase the activity of the isolated RF of Cbl-c (Fig. 6). Thus, it appears that the effects on E3 activity are a direct consequence of the binding of the LIM domain of Hic-5 to the RF of Cbl-c. In cells, Hic-5 increases Cbl-c-mediated ubiquitination of a model substrate, the EGFR (Fig. 7). This suggests that the interaction of Hic-5 with Cbl-c does not just enhance autoubiquitination by Cbl-c but also increases the E3 activity of Cbl-c towards substrates. Our previous data suggest that the phosphoryation of the linker or deletion of the N-terminus of the Cbl proteins alters the interaction between the Cbl proteins and the E2. Structures of RF/E2 complexes indicate that regions surrounding the second zinc coordinating complex interact with the loops on the E2 protein [3], [4]. Thus, Hic-5 binding is likely to occur in a region that may alter E2 binding. The proximity of the binding site of Hic-5 to the binding site for the E2 suggests that it may affect the interaction between the Cbl-c RF and the E2. Alternatively, the binding of Hic-5 to the Cbl-c RF could allow Hic-5 to interact with the E2 and induce allosteric changes that enhance ubiquitin transfer. Further studies are needed to determine the structural nature of the interaction between the LIM2 of Hic-5 and the RF of Cbl-c and to determine the mechanism underlying the enhancement of E3 activity.

To our knowledge this is the first report showing a LIM domain having a direct interaction with a RF domain. We demonstrate clearly here that this interaction contributes to the regulation of E3 activity for Cbl-c. Given the large number of RF proteins and LIM domain proteins [3], [30] it is likely other LIM proteins interact with and regulate the E3 activity of other RF proteins including the other Cbl proteins Cbl and Cbl-b.
